# Plasma mcfDNA Sequencing May Improve Usual Care Diagnostics to Detect HHV-8 Among Outpatient People with Advanced HIV

**DOI:** 10.20411/pai.v10i1.788

**Published:** 2025-02-11

**Authors:** Sarah Y. Park, Brian Epling, Morgan Richey, Daniel Lupu, Mona Mughar, Irini Sereti

**Affiliations:** 1 Medical Affairs and Clinical Affairs, Karius, Inc., Redwood City, California; 2 HIV Pathogenesis Section, Laboratory of Immunoregulation, National Institute of Allergy and Infectious Diseases; 3 Clinical Affairs, Karius, Inc., Redwood City, California

**Keywords:** Sarcoma, Kaposi, Herpesvirus 8, Human, Outpatients, HIV Infections, Cell-Free Nucleic Acids, Polymerase Chain Reaction

## Abstract

**Background:**

Human herpesvirus-8 (HHV-8), or Kaposi sarcoma (KS)-associated herpesvirus (KSHV), causes severe disease in people with profound immunosuppression. Yet, diagnosing KS can be challenging given the diverse manifestations and current limited usual care diagnostic methods (UC; polymerase chain reaction, histopathology).

**Methods:**

Pathogen-agnostic plasma microbial cell-free DNA sequencing was applied to banked samples from 116 outpatients included in 2 previous prospective studies of patients with antiretroviral treatment-naïve, advanced HIV (CD4 count ≤100 cells/μL). We then reviewed clinical and laboratory data for any people who tested positive for HHV-8 by mcfDNA sequencing or UC at baseline.

**Results:**

HHV-8 was detected in 21 (18%) outpatients with advanced HIV by any method, with males comprising the majority (86%) and one-third originally from non-US countries (including Africa, Central America, and the Caribbean). Adding mcfDNA sequencing to UC proportionally increased HHV-8 detection by 38%, while also identifying in 18 (86%) people other microbes of potential interest, including common herpesviruses, *Mycobacterium tuberculosis*, and *Pneumocystis jirovecii*.

**Conclusions:**

Plasma mcfDNA sequencing may improve UC in detection of HHV-8 infection, especially in immunocompromised outpatients, in whom early detection may facilitate appropriate management to prevent severe KS disease. The potential added benefit of the detection of other pathogens by mcfDNA sequencing may be particularly relevant for this population.

## INTRODUCTION

Human herpesvirus-8 (HHV-8), also known as Kaposi sarcoma-associated herpesvirus (KSHV), is a lymphotropic and oncogenic virus of concern for immunocompromised populations. HHV-8 seroprevalence varies globally and has been reported to be >10% in some Mediterranean countries and even >50% in parts of sub-Saharan Africa [1, 2]. In the United States, the overall seroprevalence is estimated to be much lower, from 1% to 5% [1][3]. However, the risk for HHV-8 infection is higher among men who have sex with men (MSM), with seroprevalence reported as 13% to 20%, and even higher at 30% to 35% among MSM with HIV infection [1]. While most individuals who are latently infected tend to be asymptomatic, profound immunosuppression such as with advanced HIV or post-transplantation can increase risk for developing Kaposi sarcoma (KS) or other related complications and can even lead to rapidly fatal outcomes [1, 3]. Early diagnosis of asymptomatic HHV-8 infection may therefore be preventative for at-risk populations.

HHV-8 infection and its associated conditions are challenging to diagnose given the wide variation in manifestations [1–3], often subtle symptoms, and limited available diagnostic tests. While no gold standard exists for detecting HHV-8 infection, definitive clinical diagnosis of KS must be proven by consistent histopathology findings from biopsied tumor tissue, including detection of HHV-8 by immunohistochemical staining [1, 2]. Polymerase chain reaction (PCR) of tumor tissue to detect HHV-8, although unavailable commercially, may be used also to confirm diagnosis; meanwhile, the diagnostic role of PCR to quantify HHV-8 in blood is debatable and lacks standardization [1, 4]. Similarly, while serologic testing can be useful in determining seroprevalence and population-level estimates of HHV-8 prevalence, the variability in performance and lack of standardization limit its usefulness for individual diagnosis [1, 5]. For both modalities, detection of HHV-8 viremia or seropositivity does not necessarily signify clinical disease at the time of testing.

Plasma microbial cell-free DNA (mcfDNA) metagenomic sequencing is a pathogen-agnostic approach that may offer rapid detection of HHV-8 as well as other pathogens in immunocompromised populations. We sought to determine its potential to improve usual care diagnostics (UC) to detect HHV-8 in people with advanced HIV who were antiretroviral treatment (ART) naïve.

## METHODS

### Study Design

We conducted a retrospective study of people who tested positive for HHV-8 by mcfDNA sequencing or UC at baseline, defined as within 2 weeks of enrollment in 2 previous prospective follow-up studies of people with advanced HIV disease to assess them longitudinally after ART initiation. We applied mcfDNA metagenomic sequencing to banked plasma specimens from the 116 outpatients from these 2 previous studies. In both studies, participants were adults (≥18 years) with a diagnosis of advanced HIV (CD4 count ≤100 cells/μL), were ART naïve, and were evaluated at the National Institutes of Health (NIH) between December 2006 and September 2022. They were enrolled in either Immune Reconstitution Syndrome in HIV-Infected Patients Taking Antiretroviral Therapy (IRIS protocol), NCT00286767, or PET Imaging and Lymph Node Assessment of IRIS in People with AIDS (PANDORA), NCT02147405. Participants underwent clinical evaluation, usual clinical management, laboratory studies, and research blood collection (including peripheral blood mononuclear cells, serum, and plasma). We defined UC as HHV-8 PCR of blood and histopathology of biopsied lesions. HHV-8 PCR (Quest Diagnostics Infectious Disease) of peripheral blood was not performed routinely for IRIS study participants but was collected as part of the study protocol for all PANDORA study participants. Sample type (ie, whole blood, serum, or plasma) was not standardized across all patients. Biopsy and tissue histopathology was performed when KS was suspected. Participants were followed up to 192 weeks after ART initiation under the IRIS protocol and 96 weeks after ART initiation under the PANDORA protocol. All participants provided written informed consent before study enrollment in accordance with the Declaration of Helsinki.

### Plasma Microbial Cell-free DNA Sequencing

The 116 archived plasma specimens collected during the enrollment clinical evaluation and maintained in frozen storage at −80°C under continuous monitoring were shipped to the Karius, Inc., clinical laboratory, certified under the Clinical Laboratory Improvement Amendments of 1988 and accredited by the College of American Pathologists, for plasma mcfDNA sequencing as previously described [6, 7] Sequencing data were analyzed using the Karius Test® bioinformatic pipeline version 3.16, which was designed to report mcfDNA from up to 1,000 microbes and provides the absolute plasma concentration of mcfDNA in molecules per microliter (MPM) for each microbe detected [6, 7]. As MPM values from different microbes are not comparable, a reference interval determined from a cohort of 684 healthy adult volunteers is provided in clinical result reports for comparison as an aid to interpretation [7]. Plasma mcfDNA sequencing is pathogen-agnostic for DNA microbes; polymicrobial detections especially in immunocompromised patients is possible. For this study, we focused on detections of HHV-8, although if mcfDNA sequencing yielded other microbes from a sample, the data are provided.

## RESULTS

From a combined cohort of 116 people with ART naïve, advanced HIV in the outpatient setting, we identified 21 (18%) people in whom HHV-8 was detected by any diagnostic study at baseline. Males comprised the majority (n=18, 86%) with ages ranging from 19 to 66 years (median 32 years, IQR 30–43). One third (n=7) were originally from non-US countries (including from Africa, Mexico, Central America, and the Caribbean). The median HIV viral load was 169,124 copies/mL (IQR 77,166–244,071), and the median CD4, 18 cell/mm^3^ (IQR 10–42). The median time from enrollment to ART start was 14 days (IQR 7–18).

**Figure 1.**
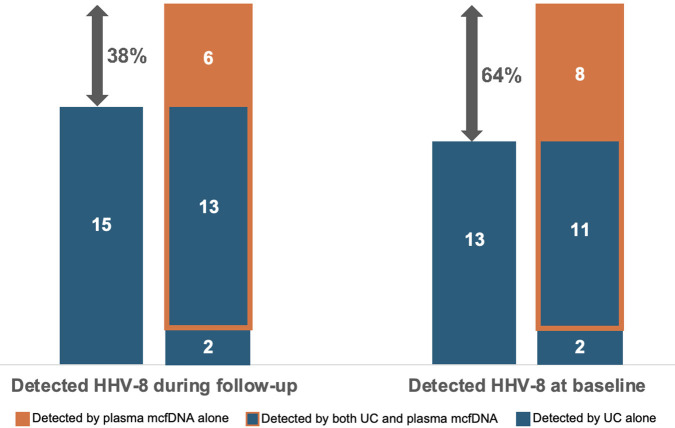
**Detection of HHV-8 (KSHV) in cohort of outpatients with advanced HIV by various diagnostic modalities.** The proportional increase in HHV-8 detections by adding plasma mcfDNA sequencing to UC (ie, blood PCR and histopathology) is shown by the double-sided arrows and percentages. Of the 116 outpatients in the study cohort, UC plus mcfDNA sequencing detected HHV-8 in 21 (18%) patients. UC alone detected 15 (13%) overall, including testing in patients who developed KS weeks after baseline; 13 (11%) when limiting to testing only at baseline. Abbreviations: HHV-8, human herpesvirus-8; KS, Kaposi sarcoma; KSHV, Kaposi sarcoma-associated herpesvirus; mcfDNA, microbial cell-free DNA; UC, usual care diagnostics

Among the 21 people in whom HHV-8 was detected by any diagnostic modality, mcfDNA sequencing detected HHV-8 in 19 (90%); MPMs ranged from 16 to 4,660 (Table 1). PCR of primarily whole blood samples detected HHV-8 in 8 (38%). Histopathology of biopsied lesions confirmed KS in 12 (57%); the other 9 (43%) did not undergo tissue biopsy for histopathology examination as they did not present with or later develop clinical manifestations of HHV-8 infection. HHV-8 was detected by mcfDNA alone in 6 (29%). Concordance and discordance in HHV-8 detection by UC versus mcfDNA and the proportional increase in yield by adding mcfDNA to UC (from 38% to as much as 64%) is demonstrated in Figure 1. Four people had concordant histopathology findings obtained during follow-up at 2, 4, 5, and 8.5 weeks, respectively, with 2 lacking any PCR testing and the other having positive HHV-8 PCR at baseline. Among those who developed KS disease, PCR detected HHV-8 in 83% (5/6) of those tested by this modality compared with 92% (11/12) detected by mcfDNA sequencing.

**Table 1.** HHV-8 (KSHV) Diagnostic Findings for Patients with Advanced HIV Enrolled in Study

Group	Patient number	Clinically compatible KS^a^ at baseline	HHV-8 PCR, copies/mL, sample type (days to result)	Histopathology HHV-8 Positive	HHV-8 detected by mcfDNA sequencing, MPM^b^	Other pathogens identified by mcfDNA sequencing, MPM
Never diagnosed with KS by NIH outpatient clinic	1	No	5,985, whole blood (4)	Not done	127	Human herpesvirus 5 (CMV), 105
	2	No	Qualitative result “Detected”, whole blood (NA^c^)	Not done	25	Human herpesvirus 2 (HSV-2), 87 Human herpesvirus 5 (CMV), 8,091 Human herpesvirus 7, 10 Human mastadenovirus D, 760 *Pneumocystis jirovecii*, 16,042
	3	No	Not detected, plasma (8)	Not done	50	*Mycobacterium tuberculosis* complex, 16 Human herpesvirus 4 (EBV), 40 Human herpesvirus 6A, 37
	4	No	Not detected, whole blood (>14)	Not done	20	Human herpesvirus 5 (CMV), 225 Human polyomavirus 6, 23 *Toxoplasma gondii*, 129
	5	No	Not detected, not specified (>14)	Not done	16	Human herpesvirus 2 (HSV-2), 26 Human herpesvirus 4 (EBV), 39
	6	No	Not done	Not done	39	*Brachyspira pilosicoli*, 308
	7	No	Not done	Not done	57	No other microbes detected
	8	No	Not done	Not done	317	Human herpesvirus 5 (CMV), 84
	9	No	<1000, whole blood (5)	Not done	Not detected	*Brachyspira pilosicoli*, 47 Human mastadenovirus D, 2,509
**Diagnosed with KS by NIH outpatient clinic at baseline**	10	Yes	36,330, whole blood (5)	Yes	73	Human herpesvirus 5 (CMV), 2,558 Primate erythroparvovirus 1 (parvovirus B19), 10,899 *Pneumocystis jirovecii*, 2,068
	11	Yes	18,922, not specified (5)	Yes	275	Human herpesvirus 5 (CMV), 48 Human polyomavirus 6, 280 Primate erythroparvovirus 1 (parvovirus B19), 14,301 *Enterocytozoon bieneusi*, 2,657
	12	Yes	6,466, plasma (8)	Yes	130	Human herpesvirus 5 (CMV), 5,382 Human polyomavirus 6, 51 *Pneumocystis jirovecii*, 318
	13	Yes	Not detected, whole blood (5)	Yes	82	*Helicobacter pylori*, 247 Human herpesvirus 5 (CMV), 4,393 *Pneumocystis jirovecii*, 33
	14	Yes	Not done	Yes	197	No other microbes detected
	15	Yes	Not done	Yes	66	No other microbes detected
	16	Yes	Not done^d^, not specified	Yes	373	*Helicobacter pylori*, 179
	17	Yes	Not done	Yes	Not detected	Human herpesvirus 5 (CMV), 654
**Diagnosed with KS during follow-up**	18	No	<1000, whole blood (13)	Yes^e^	38	Primate tetraparvovirus 1 (human parvovirus 4), 21
	19	No	Qualitative result “Detected„, serum (8)	Yes^e^	932	*Mycobacterium tuberculosis* complex, 255 Human herpesvirus 5 (CMV), 154 *Cryptosporidium hominis*, 33
	20	No	Not done	Yes^e^	4,660	Human herpesvirus 5 (CMV), 45,622
	21	No	Not done	Yes^e^	20	Human mastadenovirus B, 86

a Clinically compatible KS defined as demonstrating nontender, hyperpigmented, macular or nodular skin or mucosal lesions with or without lymphatic or visceral involvement; baseline was defined as within 2 weeks of enrollment.

b As archived specimens from the enrollment clinical evaluation were batched and tested, days to result for all plasma samples was 3.

c Time of result was not able to be determined from review of the electronic medical record report.

d PCR attempted but despite repeat analysis, non-amplification of internal control suggested presence of PCR inhibitors.

e Patient 18 was diagnosed with KS immune reconstitution inflammatory syndrome at 5 weeks after enrollment; Patient 19 was diagnosed with Castleman's at 8.5 weeks; Patient 20 had compatible symptoms triggering workup on day 31, with final pathology results on day 42; Patient 21 reported new oral lesion on day 14 prompting otolaryngology evaluation; pathology resulted on day 22.

CMV, cytomegalovirus; EBV, Epstein-Barr virus; f/u, follow-up; HSV-2, human herpesvirus 2; HHV-8, human herpesvirus 8; HIV, human immunodeficiency virus; KS, Kaposi sarcoma; KSHV, Kaposi sarcoma-associated herpesvirus; mcfDNA, microbial cell-free DNA; MPM, DNA molecules per microliter; NA, not available; NIH, National Institutes of Health.

Green cells highlight HHV-8 PCR detections and absence of KS histopathology findings; blue cells highlight presence of KS histopathology findings but no or negative HHV-8 PCR detections; yellow cells highlight presence of both HHV-8 PCR detections and histopathology findings; orange cells highlight HHV-8 detection by mcfDNA sequencing alone; and red cells highlight absence of HHV-8 detection by mcfDNA sequencing.

In addition to HHV-8, plasma mcfDNA sequencing detected other microbes in 18 (86%) people (Table 1). The most common additional microbes were other herpesviruses, especially cytomegalovirus in 11 (52%) with MPMs ranging from 48 to 45,622. Other detections of potential interest included *Mycobacterium tuberculosis* complex (n=2), *Pneumocystis jirovecii* (n=4), *Enterocytozoon bieneusi* (n=1), *Toxoplasma gondii* (n=1), *Cryptosporidium hominis* (n=1), adenovirus B (n=1) and D (n=2), and parvovirus B19 (n=2).

Turnaround time (TAT) from sample collection to result for PCR of blood took a median of 6.5 days, ranging from 4 to >14 days (exact TAT beyond >14 days could not be determined for 2 samples). Samples for mcfDNA sequencing were batched and tested with results available in 3 days similar to TAT reported previously (median 63 hours) [7]. TAT for histopathology could not be determined as preliminary findings would often be available to clinical teams at varying times.

## DISCUSSION

In this small study, plasma mcfDNA sequencing proportionally increased the overall yield for detecting baseline HHV-8 infection in outpatient people with ART naïve, advanced HIV by 64% and identified 92% of those who had or developed KS disease. Given the heightened risk among immunosuppressed people for developing severe KS and other clinical manifestations of HHV-8 infection, detecting HHV-8 requires both a high index of suspicion and accurate and rapid tests, as early recognition, preventive measures such as ART in people with HIV, and closer clinical follow-up have been demonstrated to be effective in controlling and even preventing KS manifestations [1, 8]. Our findings suggest plasma mcfDNA sequencing may improve UC detection of HHV-8 and also, as a pathogen-agnostic diagnostic test, identify other potentially important pathogens occurring among immunocompromised people.

Plasma mcfDNA sequencing notably detected HHV-8 in 4 people where PCR testing was negative, as well as one instance where PCR of the available sample was not possible because of the presumed presence of inhibitors. This would be consistent with the recently reported greater sensitivity of mcfDNA sequencing compared with PCR for detecting another type of pathogen [9]. Two of these 5 patients, for which PCR was negative or not performed, had signs of KS confirmed by histopathology. Considering the 9 total patients in whom mcfDNA or PCR detected HHV-8, but KS-related disease was not diagnosed by clinicians either at baseline or during the lengthy follow-up period (92 or 196 weeks depending on the parent protocol), subsequent ART may have played a role in preventing progression to disease, since, as already noted, controlling HIV replication through effective and consistent ART may prevent KS [8]. HHV-8 detection by PCR has been reported to fluctuate over time in an infected patient, and the amount of detectable HHV-8 by PCR in plasma has been associated with either direct KS therapy or a patient's clinical status [10]. While it is unclear whether detection and quantification by plasma mcfDNA sequencing might also fluctuate over time, our findings suggest it may provide an alternative to detecting HHV-8 when PCR is limited. This is especially important when considering detectable baseline HHV-8 DNA has been found to be predictive of KS-IRIS and associated with higher KS mortality in high-prevalence populations [11], such as those people from non-US, high HHV-8 endemic countries.

Correlation between KS and HIV viral load over CD4 count has been observed by others [10, 11], although the role of HHV-8 quantitation in blood by PCR in diagnosing clinical disease has not been established [12]. Still, the amount of HHV-8 DNA reported by PCR has been suggested as a potential marker of KS activity [10]. Whether HHV-8 mcfDNA MPM can be correlated with clinical disease or even quantitative PCR cannot be determined from the limited data of this study's small cohort, especially given not all patients underwent PCR testing for comparison. Samples from 2 patients had only a qualitative PCR result, and another 2 could only be quantified as <1000 copies per milliliter. Thus, sample size limitations suggest determining a correlation may not be as straightforward as has been demonstrated recently for another herpesvirus, cytomegalovirus [13]. One must note, too, the discordant results between mcfDNA sequencing and PCR and histopathology examination for 2 patients, respectively, which highlight gaps in understanding when HHV-8 mcfDNA is detectable. Indeed, mcfDNA detection dynamics may be impacted by unrecognized factors or biomarkers that may be better elucidated and even shed light on the pathogenesis of KS with a larger, powered sample size and a specific protocol focused on HHV-8 and development of KS.

In conclusion, our findings demonstrate the potential of plasma mcfDNA sequencing to improve UC tests in recognizing HHV-8 infection and even facilitating KS diagnosis in those with compatible clinical symptoms. Especially important for immunocompromised patients is the added value of also potentially identifying other highly relevant opportunistic infections to facilitate comprehensive care, which may offset the cost of this novel pathogen-agnostic diagnostic tool compared with target specific PCR tests. While the study cohort comprised patients with advanced HIV, our findings may also be critically relevant for solid organ transplant recipients, who are reportedly up to 200-fold more likely to develop KS relative to the general population [14]. A recent retrospective study in a single center reported that plasma mcfDNA sequencing positively impacted 1 adult SOTR for every 4 tested by identifying microbes beyond conventional testing and across syndromes [15], thus demonstrating the potential of plasma mcfDNA sequencing in this heterogeneous population. Still, as suggested for the HIV population, larger prospective studies involving the SOTR population would facilitate determining the appropriate diagnostic algorithm in determining HHV-8 infection and KS disease. When considering the HIV Organ Policy Equity (HOPE) Act, which allows for transplanting organs from deceased donors with HIV to recipients with HIV, and ongoing initiatives to increase awareness and thereby increase the donor pool as well as reduce the stigma associated with HIV [16], optimizing rapid recognition of HHV-8 infection among SOTR as well as those with advanced HIV with promising novel diagnostic technologies such as mcfDNA sequencing is especially critical.
